# Mechanisms for mechanical trapping of geologically sequestered carbon dioxide

**DOI:** 10.1098/rspa.2014.0853

**Published:** 2015-03-08

**Authors:** Yossi Cohen, Daniel H. Rothman

**Affiliations:** Lorenz Center, Department of Earth Atmospheric and Planetary Sciences, Massachusetts Institute of Technology, Cambridge, MA 02139, USA

**Keywords:** reactive transport model, carbon sequestration, Grotthuss mechanism, structural diffusion, invasion percolation, self-sealing

## Abstract

Carbon dioxide (CO_2_) sequestration in subsurface reservoirs is important for limiting atmospheric CO_2_ concentrations. However, a complete physical picture able to predict the structure developing within the porous medium is lacking. We investigate theoretically reactive transport in the long-time evolution of carbon in the brine–rock environment. As CO_2_ is injected into a brine–rock environment, a carbonate-rich region is created amid brine. Within the carbonate-rich region minerals dissolve and migrate from regions of high-to-low concentration, along with other dissolved carbonate species. This causes mineral precipitation at the interface between the two regions. We argue that precipitation in a small layer reduces diffusivity, and eventually causes mechanical trapping of the CO_2_. Consequently, only a small fraction of the CO_2_ is converted to solid mineral; the remainder either dissolves in water or is trapped in its original form. We also study the case of a pure CO_2_ bubble surrounded by brine and suggest a mechanism that may lead to a carbonate-encrusted bubble owing to structural diffusion.

## 1. Introduction

The sequestration of CO_2_ in geological formations is widely considered to be an important approach for mitigating the rise of atmospheric CO_2_ levels [1–5]. Deep saline aquifers and gas fields are primarily chosen for storage [1,4,6]. Supercritical CO_2_ is injected into these porous media while displacing another fluid, brine [5,7]. The propagation of the CO_2_ through the reservoir displays a variety of fluid-dynamical instabilities [8,9]. After injection and the cessation of buoyancy-driven flow, chemical dissolution and precipitation dominate the ensuing evolution of the reservoir. This late stage of the evolution, which remains poorly understood [10,11], is the focus of this paper.

As the CO_2_ is injected into the brine–rock environment, it initially becomes trapped, either by a physical mechanism in the presence of low permeability rocks, or by retention as a separate phase in the pore space owing to interfacial tension [12]. The disordered structure of the void spaces forces the injected fluid along certain paths that create regions, or bubbles, of the injected fluid amid regions of the defending fluid, and vice versa [13–16]. This process is known as invasion percolation when, as for the supercritical CO_2_, the invading fluid is non-wetting. For two immiscible fluids, the fluid configuration is often determined by the structure of the rock and by surface tension effects [8]. Further, the two-phase system can become unstable when reactant particles migrate from one phase to the other and change the chemical composition of each phase [10,17]. Within the high CO_2_ phase, minerals dissolve [18,19]; diffusion causes minerals and carbonate species to migrate from high-to-low concentration regions, and a fraction of them precipitates [18]. In nature, this process can be seen in hot springs, when a bubble of oxygen emerges from photosynthetic cyanobacteria [20,21]. The high gradient of CO_2_ between the bubble and the surrounding water leads to loss of CO_2_ in the vicinity of the bubble, drives up the saturation level and eventually a crust is created around the bubble. In general, mineral precipitation on a small boundary layer at the interface may lead to lower diffusivity and slower kinetics. In the carbon sequestration process, this may cause a mechanical trapping of the CO_2_ bubble and lower the solidification rate of the carbon minerals.

Here, we develop theoretical understanding of this process of mechanical phase separation. We consider two scales: at the microscale, a single CO_2_ bubble is surrounded by brine in the void space of a porous medium. In this case, the reactions occur at the interface between the bubble and the brine, as the CO_2_ dissolves and reduces the pH in its vicinity. The macroscale averages over many such bubbles. In this case, a high concentration of the invaded CO_2_ changes the properties of a macroscopic region. The region becomes more acidic, and no precipitation occurs unless the carbonate species migrate to a different region.

This paper begins by addressing the macroscale problem and the mathematical background of the reactive diffusion equation. We then study the mobility change in the fluid–rock system owing to the precipitate minerals. Finally, we consider the microscale case of a single CO_2_ bubble amid brine and suggest a second mechanism that may also lead to separation and self-sealing.

## 2. Macroscopic reactive transport

**Figure 1.**
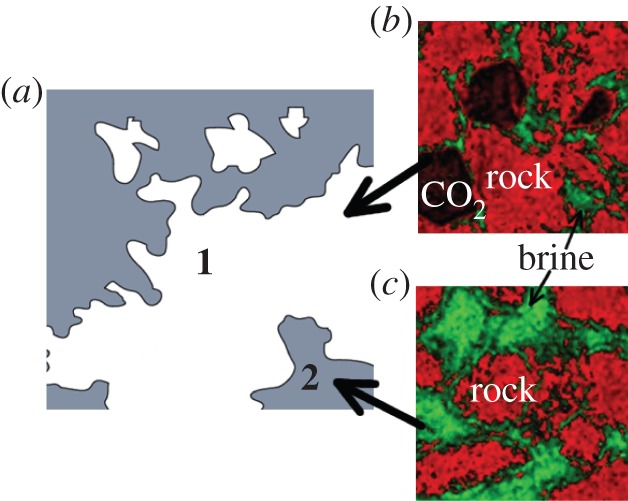
*Macroscale*: (*a*) Irregularly shaped regions of brine (grey) amid carbonate-rich regions (white) [17]. *Microscale:* CT image of material distribution in Frio sandstone, adapted from reference [25]. (*b*) Region **1** consists of CO_2_ bubbles surrounded by brine and grains of the rock. (*c*) Region **2** is the brine–rock system. (Online version in colour.)

The injection of supercritical CO_2_ (scCO_2_) into a porous medium, initially occupied with another fluid, i.e. brine (salty water), generates two different regions in the system [11,17,22]. The first region is where the CO_2_ displaced most of the existing brine. In this region, the void space in the porous medium is filled with bubbles of CO_2_ and water saturated with carbonate species. The CO_2_ dissolves rapidly in the water to reach equilibrium which results in low pH, and also a high concentration of dissolved minerals [23,24]. The second region is the intact brine–rock system, which is characterized by a low concentration of CO_2_ and higher pH. Figure 1 depicts the complexity of the CO_2_–brine–rock system.

The existence of two phases and concentration gradients drives the components to migrate from one phase to another. As they diffuse, they react to reach a local equilibrium. Although pure scCO_2_ may be clogged owing to mechanical or capillary trapping [1,26], in the presence of water, it can dissolve into its ionic forms, i.e. bicarbonate and carbonic acid, until it reaches a local thermodynamical equilibrium [27]. These carbonate species may diffuse more easily through the brine. Within the high CO_2_ phase, minerals dissolve because of the acidic environment. They then migrate from high-to-low concentration regions and a fraction of them precipitates. The evolution of each component can be described by the reactive diffusion equation

2.1 $$\frac{\partial C_{i}}{\partial t}=\nabla \cdot (D_{i}\nabla C_{i})+R_{i}(C_{1},C_{2},\ldots ,C_{5}).$$

Here, the *C*_*i*_, *i*=1,…,5 are the concentration of the ${\text{CO}}_{2},{\text{HCO}}_{3}^{-},{\text{CO}}_{3}^{2-},{\text{H}}^{+}$ and the mineral Ca^2+^, respectively. *D*_*i*_ is the isotropic diffusion coefficient for the *i*th component, and *R*_*i*_ is the reaction rate defined by the carbonate system [27,28] and the dissolution and precipitation of calcite mineral. The latter can be expressed as [29–31]

2.2 $$\frac{dM}{dt}=-k_{m}(1-\Omega ),$$

where *M* represents the density of precipitated calcite. The rate coefficient is defined by [30]

2.3 $$k_{m}=A(\text{r},t)(k_{+1}[H^{+}]+k_{+2}[{\mathrm{CO}}_{2}]+k_{+3}),$$

where *A*(**r**,*t*) is the reactive surface area, and *k*_+*i*_ are the rate constants for the forward reactions. The saturation ratio [29]

2.4 $$\Omega =\frac{\left\{{\mathrm{Ca}}^{2+}\}\{{\mathrm{CO}}_{3}^{2-}\right\}}{K_{sp}}$$

is defined by the ion activity product divided by the solubility constant of calcite. Details of the reactions and the values of the reaction constants can be found in the electronic supplementary material. Whether the calcite will precipitate or dissolve is determined by the value of *Ω*; when *Ω*<1, dissolution occurs; when *Ω*>1, mineral precipitates and when *Ω*=1, the system is at equilibrium.

## 3. Numerical simulation

In our model, we assume that there is no diffusion of the solid mineral as it nucleates and precipitates at the surface of the rock. In addition, the capillary forces between the scCO_2_ and the water prevent the mixing between the two phases; therefore, the diffusion constant of CO_2_ is also set to zero. Because the dissolution of calcite is considerably fast compared with dissolution of other minerals, we assume that, after injection, calcite dissolves quickly until it reaches equilibrium. Only new calcite that has been precipitated can be dissolved again. Thus, the reactive surface area is set to zero, as long as there is no previous precipitation of calcite. In other words, we set *A*(**r**,*t*)=0, when *M*(**r**,*t*)=0 and *Ω*(**r**,*t*)<1, and *A*(**r**,*t*)=1 otherwise.

We solve the reactive diffusion equations (2.1) and (2.2) for each component using the Galerkin finite-elements method on quadratic triangular grid with a fourth-order Runge–Kutta integration scheme. The simulation starts when the carbonate system, the pH and the dissolved calcium mineral are at local equilibrium in each phase. In region 1, the carbonate-rich brine–rock region (figure 1), we set the pH to 6 and the total dissolved inorganic carbon to 10^−2^ mol l^−1^. In region 2, we set pH=7 and the carbonate concentration at 10^−3^ mol l^−1^. We apply reflective boundary conditions far from the interface between the two regions. During the simulation, the concentrations at the boundary remain effectively constant. The simulation starts with the concentration of each one of the carbonate species and the dissolved calcium mineral at local equilibrium in each region.

**Figure 2.**
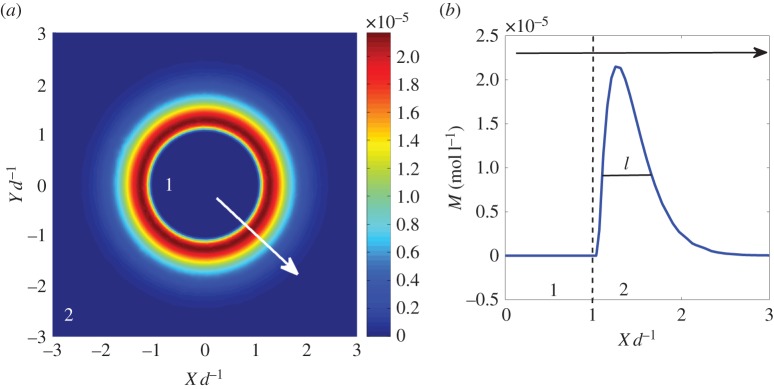
(*a*) Mineral precipitation on the periphery of a circular domain (*d*= 2 *cm*). In the inner shape is the carbonate-rich phase (region 1) which consists of high concentration of CO_2_, amid region 2, the brine. Precipitation of minerals occurs at the interface that separates the two regions. The colour indicates the accumulation of precipitated minerals. The lengths are normalized by *d*, a characteristic length of region 1, and given in dimensionless units. (*b*) The profile of the accumulated mineral at *t*=0.5 *d*^2^/*D*. The dashed line separates the two regions and the black arrow corresponds to the white arrow in (*a*). (Online version in colour.)

We consider a carbonate-rich circular shape (region 1) surrounded by brine (region 2). We find that the accumulation of calcite, shown in figure 2*a*, occurs on a small boundary layer close to the interface. The precipitation profile is approximated by the typical diffusion length, $l∼\sqrt{Dt}$, shown in figure 2*b*. However, when the contribution of the reaction terms becomes significant, we see deviations from this profile. To approximate the time scale over which this profile holds, we consider the reactive diffusion equation, equation (2.1), and the accumulation of precipitated mineral in equation (2.2). Changing equation (2.1) into dimensionless variables, x→ξd, $t\to (d^{2}/D)\tau$, leads to

3.1 $$\frac{\partial C_{i}}{\partial \tau }=\frac{\partial^{2}C_{i}}{\partial \xi^{2}}+\frac{d^{2}}{D}R_{i}(C_{j}),\;j=1,\ldots ,5.$$

where *d* is the effective domain size of region 1 that participates in the dynamics. From equation (2.2), the density of the precipitated mineral becomes

3.2 $$\frac{dM}{d\tau }=-\frac{k_{m}d^{2}}{D}\left(1-\frac{A(\tau )B(\tau )}{K_{sp}}\right),$$

where *A*(*τ*) and *B*(*τ*) correspond to the concentration of Ca^2+^ and ${\mathrm{CO}}_{3}^{2-}$. As long as *A*(*τ*) and *B*(*τ*) are purely diffusion-controlled, the system is self-similar, and the precipitation profile (figure 2*b*) holds. When the reaction term becomes dominant, a deviation from this profile will be obtained. From the numerics, the concentration of ${\mathrm{CO}}_{3}^{2-}$ is likely to deviate first from this behaviour after *τ*∼*d*^2^/*D* owing to its low concentration in the brine. The kinetics are described in detail in the electronic supplementary material.

**Figure 3.**
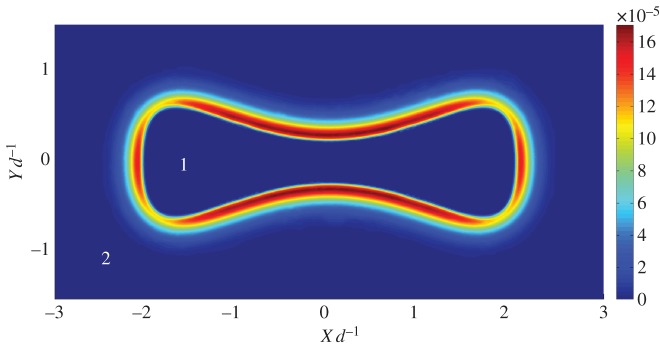
The same as figure 2*a*, but with an interface of variable curvature. Precipitation is greatest in regions of high negative curvature (concave outwards). (Online version in colour.)

Figure 3 shows qualitatively how the interface curvature alters the mineral precipitation. We find that the accumulated crust is highly dependent on the curvature of the interface owing to the nature of a diffusion process through a curved interface [32]. Initially, accumulation of mineral is effectively diffusion-controlled. Thus, negative curvature with respect to the inner bubble of the CO_2_ phase has maximum mineral precipitation at the interface, owing to the convergence of lines of flux [32,33].

Collectively, these results indicate that precipitation of mineral occurs on a small boundary layer at the interface and a carbonate crust, created in a small boundary layer, can lead to a mechanical separation of the two phases.

## 4. System size dependence

**Figure 4.**
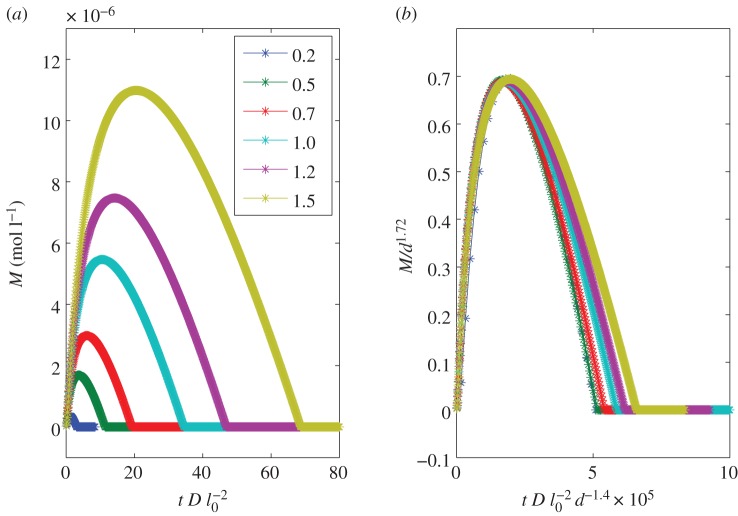
(*a*) Temporal dependence of the precipitated mineral at the interface for different system size, *d*, given in the legend in millimetres. The length *l*_0_= 1 mm. (*b*) Collapse of the data shown on the left side by rescaling the mineral density and time. The exponents are chosen such the maximum mineral density for different bubble sizes occurs at the same position in the rescaled coordinates. (Online version in colour.)

**Figure 5.**
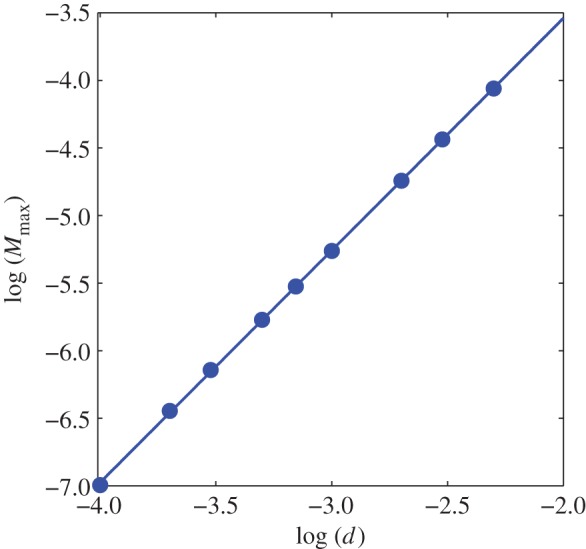
Maximum density of precipitated mineral with respect to system size. The slope is *n*=1.72. (Online version in colour.)

Mineral precipitation along the interface changes the porosity of the rock, decreases the effective diffusivity, and may clog existing voids [1,26]. The decrease in the mobility of the ions in the solution could bring further solidification of minerals to a halt. Whether a pathway between pores will be clogged or not is highly dependent on the local density of the accumulated mineral, which depends also on the amount of the carbonate species, and the size and the shape of the domain noted as region 1. To study how a change in the size alters the maximum mineral density, we initiate a one-dimensional domain of size *d* of region 1, and run the simulation until equilibrium. During the simulation, the mineral density reaches a maximum and then decreases as the solution at the interface become more acidic, shown in figure 4. We calculate the maximum calcite concentration *M* as a function of *d*. We obtain a power law relationship *M*∝*d*^*n*^, where *n*≃1.72, as shown in figure 5.

The exponent *n* can be approximated by a simple scaling analysis. If *A*(*τ*) and *B*(*τ*) in equation (3.2) are purely diffusion-controlled, i.e. if the reaction term in equation (3.1) can be neglected, the accumulated precipitation scales like *d*^2^. In practice, the exponent may be lower than 2 because of the contribution of the reaction terms that change the equilibrium state in our system.

## 5. Effective diffusivity

The diffusivity in a porous medium depends on several factors which are related to the pore geometry and the effective porosity accessible by diffusion [34,35]. For the sake of simplicity, we consider the permeability reduction to be linear with the porosity loss and mineral precipitation, and the effective diffusivity *D*_*e*_ to be linear with the porosity *ϕ*_*t*_:

5.1 $$D_{e}\propto \phi_{t}D_{0},\;\phi_{t}=\left(1-\frac{\theta }{\theta_{c}}\right),$$

where *D*_0_ is the bulk diffusivity corresponding to the initial porosity, *ϕ*_*t*_ is the porosity available for transport, *θ*=*M* is the maximum density of the precipitated mineral and *θ*_*c*_ is a critical accumulated density in which *D*_*e*_ vanishes.

**Figure 6.**
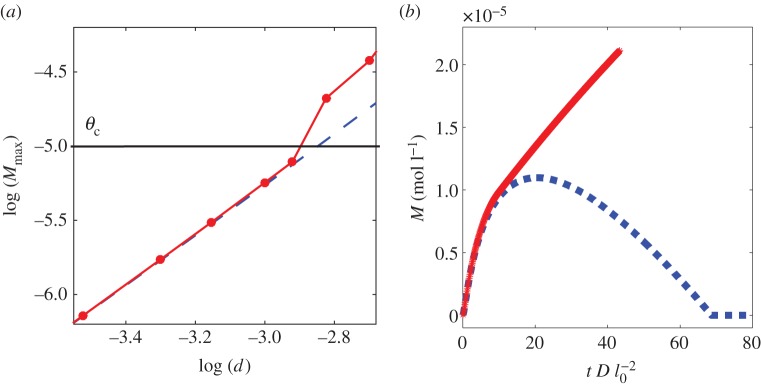
(*a*) Maximum density of precipitated mineral *M* with respect to system size *d*, with clogging. *Dashed line*: the same as figure 5 with *D*_*e*_=*D*_0_. *Solid line*: the results allowing the porosity to decrease and the effective diffusivity *D*_*e*_ to decrease as the precipitation grows. Note the enhanced precipitation when the critical accumulated density, *θ*_*c*_, is reached. At this point, particles cannot cross between the regions, and the system seeks a local chemical equilibrium. (*b*) Temporal dependence of precipitated minerals. *Dashed line*: the same as in figure 4. *Solid line*: with clogging. Note that after the diffusivity is reduced to zero, around *t*∼12, the two regions are separated and the system seeks local chemical equilibrium. Then, the dynamics stop. (Online version in colour.)

As the accumulated mineral reaches the critical porosity, it changes locally the permeability and mechanical separation may occur owing to diffusivity loss and self-sealing. We also observe that while the diffusion coefficient decreases, it creates even more precipitation in a smaller area close to the interface. These results, shown in figure 6, are in agreement with the diffusion length scale of $l∼\sqrt{D_{e}t}$ from the numerical approximation and the mineral precipitation of *M*∼1/*D*_*e*_ from equation (3.2). In addition, when the system is clogged, i.e. when the effective diffusivity goes to zero, the system seeks a local chemical equilibrium.

From the numerical results, we can identify two trapping mechanisms that occur as particles migrate from one region to the other. For small carbonate-rich regions, the CO_2_ dissolves completely into the brine, and the low pH does not allow a significant precipitation of the carbonate minerals. Thus, the CO_2_ is trapped in its dissolved forms. For larger regions, solidification of minerals stops as clogging occurs, and the developed crust separates the two regions. In both cases, the total amount of the precipitated carbonate minerals is small compared with concentrations of the other carbonate species.

## 6. The microscale: a single carbon dioxide bubble

**Figure 7.**
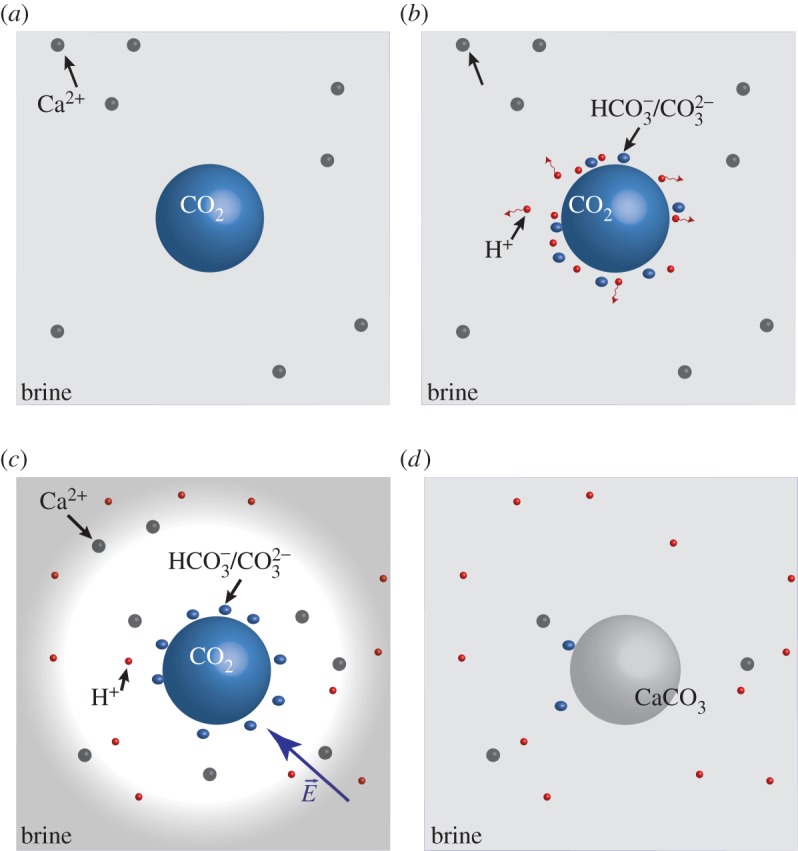
Evolution of a CO_2_ bubble (not in scale): (*a*) a single bubble of pure CO_2_ surrounded by brine. (*b*) At the interface, the CO_2_ dissolves into the brine, and reduces the pH. (*c*) Protons migrate faster from the interface, creating an electrostatic dipole and attracting minerals towards the interface. (*d*) Minerals precipitate owing to supersaturation, and a crust is created. (Online version in colour.)

Here, we discuss a mechanism that leads to a carbonate-encrusted bubble in which a single bubble of pure CO_2_ surrounded by brine develops a crust at the interface. Unlike the upscaled case discussed above, where each region consists of the same ingredients but with a different concentration, here we have a pure bubble (no upscaling) with only CO_2_, either in a liquid phase or gas phase. The problem is pictured in figure 7. In this case, no chemical reactions occur inside the CO_2_ bubble; reactions instead occur only at the interface with the brine. The CO_2_ then dissolves into the brine, creates charged carbonate species and reduces the pH in its vicinity. Most of the components diffuse slowly away from the interface; however, the protons in water diffuse faster owing to structural diffusion, also known as the Grotthoss mechanism [36]. The concentration gradient with unequal diffusivities generates an electrical field that slows down the rapidly diffusing protons, but also attracts positively charged minerals towards the interface. (The dynamics is described by the Nernst–Planck equations [37,38]). This leads to supersaturation at the interface and mineral precipitation. This process suggests that an isolated bubble can remain stable owing to a self-sealing mechanism. The actual determination whether a bubble will be encrusted or not depends on the environment and the dynamical behaviour of the bubble. First, the velocity of the bubble should be effectively zero or smaller than the mobility of the ions in the solution in order to build the electrostatic field in the vicinity of the bubble. Second, the amount of mineral cations that are attracted to the interface owing to the electrostatic field should suffice to reach supersaturation that causes precipitation. Third, to seal a bubble, the field must be homogeneous in order to create a uniform and stable crust. A quantitative measure of the conditions that lead to self-sealing could be obtained by experiment.

## 7. Discussion and conclusion

We have shown that in a system of reactive fluids, a gradient in the concentration between regions leads to a supersaturation at the interface and to precipitation and porosity loss. A local change in the porosity reduces permeability and may cause mechanical separation. This process is important in understanding the long-term sequestration of carbon dioxide in subsurface geological formation. We predict that a small domain of a region consisting mostly of the carbonate species in an acidic environment will dissolve completely into the brine. Larger domains are more likely to be self-sealed, and only a fraction of the carbonate species will be precipitated. For a single CO_2_ bubble in brine solution, we suggest a mechanism that causes self-sealing owing to the pH gradient at the interface of the bubble and structural diffusion.

Our results suggest that only a small fraction of the injected CO_2_ is converted to a solid mineral. The remainder stays in its dissolved ionic form or is trapped in its original form. Whether a domain will go into dissolution trapping or mechanical separation depends on the concentration gradients, the properties of the rock and the porosity available for transport.

## Supplementary Material

A numerical model for the CO2 sequestration

## Data accessibility

The data supporting this article have been uploaded as part of the electronic supplementary material.
