# Supplementary material for: Mechanisms for mechanical trapping of geologically sequestered carbon dioxide
Source: Proc Math Phys Eng Sci. 2015 Mar 8;471(2175):20140853. doi: 10.1098/rspa.2014.0853 (PMC4353055; doi:10.1098/rspa.2014.0853)
Supplement: A numerical model for the CO2 sequestration [file rspa20140853supp1.pdf]

A numerical model for the CO_2 sequestration

Yossi Cohen and Daniel H. Rothman

I. REACTIONS

In the numerical model, we consider the following reactions: The carbonate system is given by

$$CO_2 + H_2O \underset{k_{-1}}{\overset{k_{+1}}{\rightleftharpoons}} HCO_3^- + H^+ \underset{k_{-2}}{\overset{k_{+2}}{\rightleftharpoons}} CO_3^{2-} + 2H^+, \quad (1)$$

here k_{+i} and k_{-i}, $i = 1, 2$ are the reaction constant rates for the forward and backward reactions, respectively. The values of the reaction constants are taken from [1]: $k_{+1} = 0.037$ s^{-1}, $k_{-1} = 2.66 \cdot 10^4$ kg/(mol·s), $k_{+2} = 59.4$ s^{-1} and $k_{-2} = 5 \cdot 10^{10}$ kg/(mol·s).

In the model, we consider the evolution of the mineral calcium carbonate and its reaction, as representative of the precipitation process. While other minerals affect the carbonate system, calcium carbonate is one of the most important reactive ingredients. It also has very rapid kinetics that are greater than 5 to 6 order of magnitude than the rates of dissolution of other minerals, such as plagioclase (cf. [2], [3] and references therein). The dissolution of calcium carbonate occurs via three parallel reactions [5]:

$$CaCO_3 + H^+ \overset{k_4}{\rightleftharpoons} Ca^{2+} + HCO_3^-, \quad (2)$$

$$CaCO_3 + CO_2 + H_2O \overset{k_5}{\rightleftharpoons} Ca^{2+} + 2HCO_3^-, \quad (3)$$

$$CaCO_3 \overset{k_6}{\rightleftharpoons} Ca^{2+} + CO_3^-. \quad (4)$$

The rate of precipitation and dissolution of carbon mineral (reactions 2, 3 and 4) can be written as [3, 4]

$$R = A\left(k_4\{H\} + k_5\{CO_2\} + k_6\right)(1 - \Omega) \quad (5)$$

where

$$\Omega = \{Ca^{2+}\}\{CO_3^{2-}\}/K_{sp}. \quad (6)$$

$K_{sp} = 4.265 \cdot 10^{-7}$ is the solubility product or the solubility equilibrium of the reaction; A is the reactive surface area; and $k_4 = 8.9 \cdot 10^{-5}$, $k_5 = 5 \cdot 10^{-8}$ and $k_6 = 6.5 \cdot 10^{-11}$ are the

rate constants for the forward reaction in mol/(cm$^2\cdot$ s). For simplicity, we assume that the activity of a species, $\{\cdot\}$, is equal to its concentration, $[\cdot]$.

The pH and the total dissolve inorganic carbon (DIC) are specified in each region. The simulation starts when the carbonate species and the calcium mineral are at local equilibrium. The concentration values are found according to [1]

$$[\mathrm{CO_2}] = \mathrm{DIC} \Bigg/ \left(1 + \frac{K_1}{[\mathrm{H^+}]} + \frac{K_1 K_2}{[\mathrm{H^+}]^2}\right), \tag{7}$$

$$[\mathrm{HCO_3^-}] = \mathrm{DIC} \Bigg/ \left(1 + \frac{[\mathrm{H^+}]}{K_1} + \frac{K_2}{[\mathrm{H^+}]}\right), \tag{8}$$

$$[\mathrm{CO_3^{2+}}] = \mathrm{DIC} \Bigg/ \left(1 + \frac{[\mathrm{H^+}]}{K_2} + \frac{[\mathrm{H^+}]^2}{K_1 K_2}\right). \tag{9}$$

and with $\Omega = 1$.

Each component evolves according to the following dynamics,

$$\frac{\partial[\mathrm{CO_2}]}{\partial t} = -k_1[\mathrm{CO_2}] + k_{-1}[\mathrm{H^+}][\mathrm{HCO_3^-}] - Ak_5[\mathrm{CO_2}](1-\Omega) \tag{10}$$

$$\begin{aligned}\frac{\partial[\mathrm{HCO_3^-}]}{\partial t} &= D\nabla^2[\mathrm{HCO_3^-}] + k_1[\mathrm{CO_2}] - k_{-1}[\mathrm{H^+}][\mathrm{HCO_3^-}] - k_2[\mathrm{HCO_3^-}] + k_{-2}[\mathrm{H^+}][\mathrm{CO_3^{2-}}] \\ &\quad + A(k_4[\mathrm{H^+}] + 2K_5[\mathrm{CO_2}])(1-\Omega)\end{aligned} \tag{11}$$

$$\frac{\partial[\mathrm{CO_3^{2-}}]}{\partial t} = D\nabla^2[\mathrm{CO_3^{2-}}] + k_2[\mathrm{HCO_3^-}] - k_{-2}[\mathrm{H^+}][\mathrm{CO_3^{2-}}] + Ak_6(1-\Omega) \tag{12}$$

$$\begin{aligned}\frac{\partial[\mathrm{H^+}]}{\partial t} &= D\nabla^2[\mathrm{H^+}] + k_1[\mathrm{CO_2}] - k_{-1}[\mathrm{H^+}][\mathrm{HCO_3^-}] + k_2[\mathrm{HCO_3^-}] - k_{-2}[\mathrm{H^+}][\mathrm{CO_3^{2-}}] \\ &\quad - Ak_4[\mathrm{H^+}](1-\Omega)\end{aligned} \tag{13}$$

$$\frac{\partial[\mathrm{Ca^{2+}}]}{\partial t} = D\nabla^2[\mathrm{Ca^{2+}}] + A\left(k_4[\mathrm{H^+}] + k_5[\mathrm{CO_2}] + k_{+6}\right)(1-\Omega) \tag{14}$$

$$\frac{\mathrm{d}[\mathrm{CaCO_3}]}{\mathrm{dt}} = -A\left(k_4[\mathrm{H^+}] + k_5[\mathrm{CO_2}] + k_{+6}\right)(1-\Omega). \tag{15}$$

Here $D = 10^{-8}$ m^2/s is the diffusion coefficient in the porous media. In section 5 in the paper, D is replaced by the effective diffusivity, D_e. We note that the carbonate system is a natural buffer for the pH [1]. Thus, the evolution of the pH is mainly defined by the rapid kinetics of the carbonate system.

---

[1] Zeebe RE, Wolf-Gladrow D. 2001 *CO_2 in Seawater: Equilibrium, Kinetics, Isotopes: Equilibrium, Kinetics, Isotopes.* Elsevier.

[2] A. F. White and S. L. Brantley 1995 *Chemical Weathering Rates of Silicate Minerals, vol. 31* Mineralogical Society of America.

[3] Li L, Steefel CI, Yang L. 2008 Scale dependence of mineral dissolution rates within single pores and fractures. Geochimica et Cosmochimica Acta. **72**(2):360–377.

[4] Chou L, Garrels RM, Wollast R. 1989 Comparative study of the kinetics and mechanisms of dissolution of carbonate minerals. Chemical Geology. **78**(3):269–282.

[5] Plummer LN, Wigley TML, Parkhurst DL 1978 Critical Review of the Kinetics of Calcite Dissolution and Precipitation Am. J. Sci. **278** 179.
